# PDCD5 inhibits progression of renal cell carcinoma by promoting T cell immunity: with the involvement of the HDAC3/microRNA-195-5p/SGK1

**DOI:** 10.1186/s13148-022-01336-1

**Published:** 2022-10-20

**Authors:** Shu-cheng Liu, Li-bo Chen, Ping-feng Chen, Meng-long Huang, Tian-pei Liu, Jun Peng, Xin-sheng Lu

**Affiliations:** 1 The First Affiliated Hospital, Department of Urology, Hengyang Medical School, University of South China, Hengyang, 421001 Hunan China; 2 The First Affiliated Hospital, Department of Neurology, Hengyang Medical School, University of South China, Hengyang, 421001 Hunan China

**Keywords:** Programmed cell death 5, Histone deacetylase 3, microRNA-195-5p, Serum glucocorticoid-inducible kinase 1, Renal cell carcinoma

## Abstract

**Background:**

Epigenetics exerts a vital role in the onset and development of renal cell carcinoma (RCC). Mounting evidence has shed light on the significance of human immune system in response to tumor infiltrating T cells. Hereby, we sought to unmask the immunomodulatory role of histone deacetylase 3 (HDAC3) and its potential upstream molecule, programmed cell death 5 (PDCD5) in RCC.

**Methods:**

RCC and adjacent non-cancerous tissues were clinically resected from 58 patients, in which the expression profile of microRNA-195-5p (miR-195-5p), PDCD5, HDAC3, and serum glucocorticoid-inducible kinase 1 (SGK1) was determined by RT-qPCR and Western blot analysis. Their relations were investigated by a series of luciferase assays in combination with ChIP and co-IP. RCC cells (A498) were intervened using gain- and loss-of-function approaches, followed by cell proliferation evaluation. After co-culture with CD3^+^ T cells, flow cytometry and interferon-*γ* (IFN-*γ*) determination were performed. A xenograft tumor mouse model was developed for in vivo validation.

**Results:**

PDCD5 was downregulated in RCC tissues and A498 cells. Upregulation of HDAC3, as well as of SGK1, resulted in suppression of A498 cell proliferation and promotion of T cell activation as evidenced by higher IFN-*γ* expression. Re-expression of PDCD5 downregulated HDAC3, causing a subsequent upregulation of miR-195-5p, while miR-195-5p could inversely modulate its target gene, SGK1. The regulatory mechanism appeared to be functional in vivo.

**Conclusion:**

Our results highlight the possible manipulation by PDCD5 on RCC cell proliferation and T cell activation, which provides new clues to better understand the immune balance in RCC progression.

**Supplementary Information:**

The online version contains supplementary material available at 10.1186/s13148-022-01336-1.

## Background

Kidney cancer is considered as the most prevalent malignancy on a global scale with 403,262 new cases and 175,098 deaths in 2018 [1]. Renal cell carcinoma (RCC) represents more than 90% of kidney cancer, which is originated from the renal epithelium tumor and characterized by more than 10 histological and molecular subtypes [2]. Upon diagnosis, the majority of patients demonstrate the symptoms of locally or localized advanced RCC, and some patients (after surgery) remain at risk of fatal metachronous distant metastases [3]. Based on these facts, there remains an urgency for developing effective treatment modalities. Intriguingly, immunity has been increasingly recognized to bear therapeutic significance to kidney cancer [4].

It is widely recognized that epigenetics plays a significant role in affecting the initiation and development of RCC [5], wherein the acetylation modification of histone mainly depending on histone acetylases and deacetylases constitutes an important mechanism to modulate the T cell-mediated immunity [6]. Therefore, intervening the histone acetylation may affect the immune response of T cells, whereby altering the occurrence and development of RCC. It has been found that histone deacetylases HDAC1 and 2 are essential for the development of RCC [7], and that histone deacetylase inhibitors hold promise as an appealing strategy for the management of RCC [8, 9]. Besides, HDAC3 has also been proposed as a promising therapeutic target for clinical management for BRM-negative clear cell RCC [10]. Notably, HDAC3 has been suggested to induce the reduction of microRNA-195-5p (miR-195-5p) in hepatocellular carcinoma, which may suggest the role of miRNA biogenesis in malignant cells [11]. Intriguingly, the upregulation of miR-195-5p has been unveiled to decelerate RCC cell growth and accelerate apoptosis, thus curbing the progression of RCC [12]. Of note, serum glucocorticoid-inducible kinase 1 (SGK1) is considered as a key to the balance between regulatory T cells and T-helper cell 17 [13], and it has been elaborated to foreshadow dismal oncologic outcomes of patients with non-small cell lung cancer [14]. Interestingly, our bioinformatics analysis has revealed serum SGK1 as a putative target of miR-195-5p. Hence, it is speculated that HDAC3/miR-195-5p/SGK1 might participate in regulation of the immune response in RCC.

Programmed necrosis, processes of programmed cell death (PDCD), and apoptosis are well-known vital players of the immune system [15]. The role of PDCD5 (a pro-apoptotic protein) has been demonstrated in immunomodulation by contributing to the functions of regulatory T cells [16]. PDCD5 has been documented to have a vital regulatory role in multiple malignancies, including osteoclastoma, colon cancer, and prostate cancer [17–19]. It should be noted that PDCD5 can induce the degradation of HDAC3 [20]. Herein, given the aforementioned findings, we might hypothesize that PDCD5 could be implicated in the progression and development of RCC from the perspective of immunomodulation via the possible regulatory mechanism involving HDAC3/miR-195-5p/SGK1 axis.

## Results

### Overexpression of PDCD5 inhibits RCC cell proliferation and promotes T cell activation

**Fig. 1:**
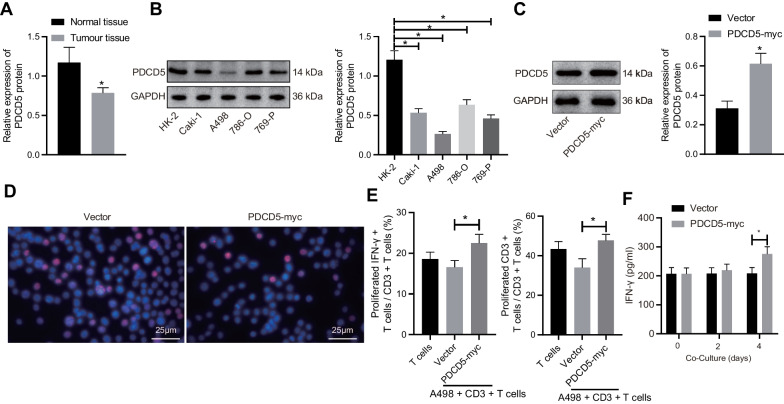
PDCD5 is under-expressed in RCC tissues and cells. **A** The expression of PDCD5 in RCC and adjacent non-cancerous tissues determined by Western blot analysis (*n* = 58). *p < 0.05 versus the adjacent non-cancerous tissues. **B** The expression of PDCD5 in RCC cell lines (Caki-1, A498, 786-O, and 769-P) and normal renal cell line (HK-2) normalized to GAPDH determined by Western blot analysis. **C** The expression of PDCD5 in RCC cells normalized to GAPDH determined by Western blot analysis to confirm the transfection efficiency. *p < 0.05 versus the vector-treated cells. **D** A498 cell proliferation following PDCD5 overexpression detected by EdU assay. **E** IFN-*γ*^+^ T cell proportion and CD3^+^ T cell proliferation following PDCD5 overexpression detected by flow cytometry. **F** The expression of IFN-*γ* released from CD3^+^ T cells following PDCD5 overexpression determined by ELISA. *p < 0.05 between the two groups. The results were measurement data and expressed as mean ± standard deviation. The results were collected from at least 3 independent cell experiments

To explore the immune regulatory role of PDCD5 in RCC, the expression profile of PDCD5 in RCC tissues and cells was firstly determined by Western blot analysis. Our results exhibited a significant downregulation of PDCD5 in RCC tissues and RCC cells (Caki-1, A498, 786-O, and 769-P) as compared to corresponding adjacent non-cancerous tissues and normal renal cells (HK-2), respectively. The most significantly downregulated PDCD5 expression was found in A498 cells. Thus, A498 cells were selected for subsequent use in cellular function assessments (Fig. 1A, B). For investigation regarding the effects of PDCD5 on RCC cell proliferation, we upregulated the PDCD5 expression using PDCD5 overexpression vector in A498 cells (Fig. 1C), followed by cell proliferation assessment using EdU assay (Fig. 1D). It was found that A498 cell proliferation was suppressed by PDCD5 overexpression.

Subsequently, A498 cells overexpressing PDCD5 were further co-cultured with CD3^+^ T cells, followed by flow cytometric analysis to assess CD3^+^ T cell proliferation and IFN-*γ*^+^ T cell proportion. Our results exhibited that both CD3^+^ T cell proliferation and IFN-*γ* T cell proportion were promoted upon PDCD5 upregulation (Fig. 1E). Meanwhile, ELISA results demonstrated that the expression of IFN-*γ* released from CD3^+^ T cells was upregulated following PDCD5 gain of function (Fig. 1F). Taken together, our data speculated that RCC proliferation was suppressed, whereas T cell activation in RCC was promoted by PDCD5 elevation.

### HDAC3 is involved in the modulatory effect of PDCD5 on RCC cell proliferation and T cell activation

**Fig. 2:**
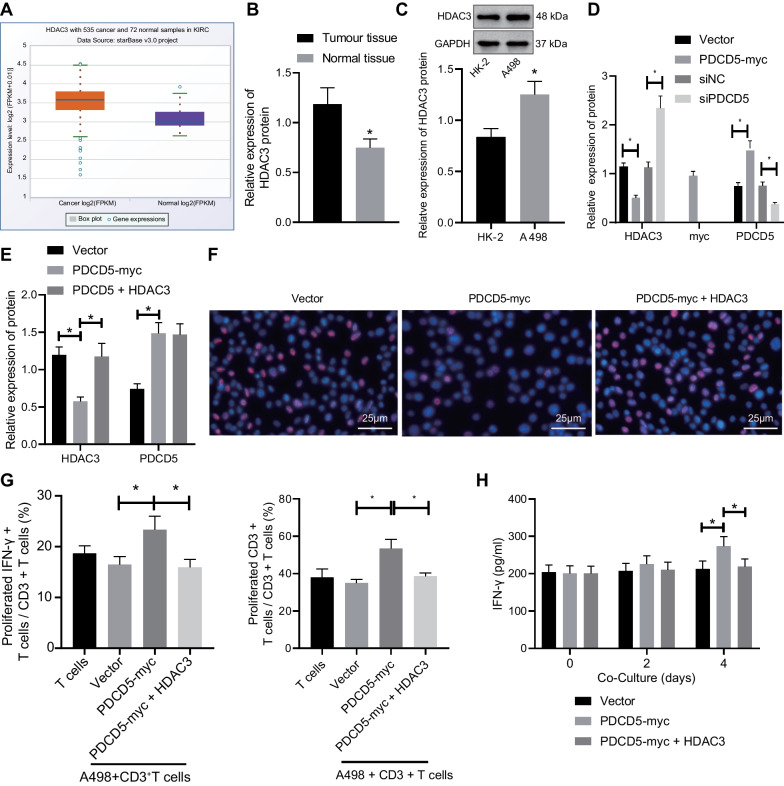
RCC cell proliferation is inhibited, and T cell activation is promoted by upregulated PDCD5 via HDAC3. **A** The expression box plot of HDAC3 in RCC samples and normal samples predicted by StarBase database. **B** The expression of HDAC3 in RCC and adjacent non-cancerous tissues determined by Western blot analysis (*n* = 58). *p < 0.05 versus the RCC tissues. **C** The expression of HDAC3 in RCC cell line (A498) and normal renal cell line (HK-2) normalized to GAPDH determined by Western blot analysis. *p < 0.05 versus the HK-2 cell line. **D** The expression of HDAC3 in A498 cells after upregulation/downregulation of PDCD5 normalized to GAPDH determined by Western blot analysis. *p < 0.05 between the two groups. **E** The transfection efficiency of PDCD5 and HDAC3 validated by Western blot analysis. *p < 0.05 between the two groups. **F** A498 cell proliferation detected by EdU assay. **G** IFN-*γ*^+^ T cell proportion and CD3^+^ T cell proliferation detected by flow cytometry. **H** The expression of IFN-*γ* released from CD3^+^ T cells determined by ELISA. *p < 0.05. The results were measurement data and expressed as mean ± standard deviation. The results were collected from at least 3 independent cell experiments. Data between RCC and adjacent non-cancerous tissues were analyzed by paired *t* test and data comparison between the other two groups was analyzed by unpaired *t* test

Consistent with the previous finding [20], we found through TCGA database a significant negative correlation between PDCD5 expression and HDAC3 expression (Additional file 1: Fig. S1A). Based on the StarBase database, HDAC3 was upregulated in RCC tissue samples (Fig. 2A). Coherently, our Western blot results revealed a significantly upregulated expression of HDAC3 in RCC tissues (Fig. 2B) and A498 cells (Fig. 2C) in comparison with their corresponding controls.

To further testify the correlation between HDAC3 and PCDC5, PCDC5 was upregulated by PDCD5 overexpression vector or downregulated by siRNA against PDCD5 (si-PDCD5) in A498 cells. The results substantiated an inverse relationship between HDAC3 and PCDC5 in RCC (Fig. 2D). Furthermore, endogenous co-IP assay showed that PDCD5 selectively interacted with HDAC3 among class I HDACs (Additional file 1: Fig. S1B), suggesting HDAC3 as a unique PDCD5-binding protein in class I HDACs. In addition, silencing of PDCD5 significantly reduced the HDAC3 ubiquitination (Additional file 1: Fig. S1C).

After that, we upregulated HDAC3 in the PDCD5-abundant A498 cells to evaluate whether HDAC3 might underlie the modulatory effect of PDCD5 on RCC cell proliferation and T cell activation. Western blot analysis validated the transfection efficiency of overexpression of PDCD5 and HDAC3 (Fig. 2E). EdU assay demonstrated that overexpression of HDAC3 restored the suppressed RCC cell proliferation induced by overexpressed PDCD5 (Fig. 2F). Then, flow cytometric analysis (Fig. 2G) and ELISA data (Fig. 2H) revealed that additional HDAC3 overexpression inhibited the promoting effects of PDCD5 overexpression on T cell activation and the expression of CD3^+^ T cell-released IFN-*γ*. Collectively, these above-described findings indicated that PDCD5 inhibited RCC cell proliferation and promoted T cell activation by silencing HDAC3 expression.

### HDAC3 silencing facilitates miR-195-5p transcription to impede RCC cell proliferation and stimulate T cell activation

**Fig. 3:**
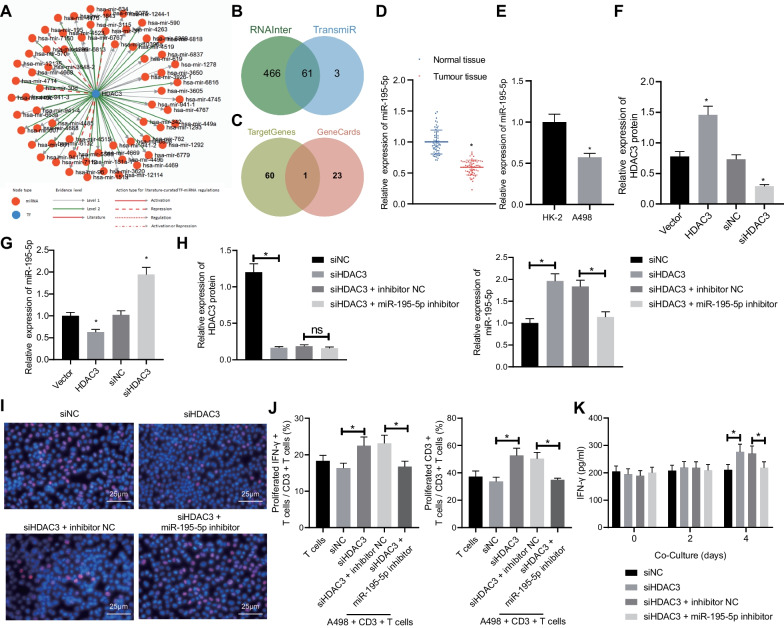
RCC cell proliferation is suppressed, and T cell activation is enhanced by silencing HDAC3 via miR-195-5p. **A** miRNAs targeted by HDAC3 determined with TransmiR v2.0 website. **B** Venn diagram of Intersection of miRNAs targeted by HDAC3 predicted with TransmiR v2.0 and RNAInter. **C** Venn diagram of RCC-related miRNAs and candidate targeted miRNAs in the GeneCards database. **D** miR-195-5p expression in RCC and adjacent non-cancerous tissues determined by RT-qPCR (*n* = 58). *p < 0.05 versus the adjacent non-cancerous tissues. **E** miR-195-5p expression in RCC cell line (A498) and normal renal cell line (HK-2) determined by RT-qPCR. *p < 0.05 versus the HK-2 cell line. **F** The expression of HDAC3 in A498 cells normalized to GAPDH determined by Western blot analysis. *p < 0.05 versus the vector or si-NC group. **G** miR-195-5p expression in A498 cells after overexpression or silencing of HDAC3 determined by RT-qPCR. *p < 0.05 versus the vector or si-NC group. **H** The expression of HDAC3 in A498 cells normalized to GAPDH determined by Western blot analysis and miR-195-5p expression determined by RT-qPCR after transfection. *p < 0.05 between the two groups. **I** A498 cell proliferation detected by EdU assay. **J** IFN-*γ*^+^ T cell proportion and CD3^+^ T cell proliferation detected by flow cytometry. *p < 0.05 between the two groups. **K** The expression of IFN-*γ* released from CD3^+^T cells determined by ELISA. *p < 0.05 between the two groups. The results were measurement data and expressed as mean ± standard deviation. The results were collected from at least 3 independent cell experiments. Data between RCC and adjacent non-cancerous tissues were analyzed by paired *t* test, and data comparison between the other two groups was analyzed by unpaired *t* test

The downstream miRNAs mediated by HDAC3 were obtained using bioinformatics website TransmiR v2.0 (Fig. 3A). Meanwhile, 527 miRNAs targeted by HDAC3 were obtained from RNAInter database. The intersection was then taken between the miRNAs attained from the two databases, which yielded 61 miRNAs (Fig. 3B). Subsequently, 24 RCC-related miRNAs with high correlation scores were obtained from the GeneCards database and then intersected with the aforesaid 61 candidate miRNAs. Accordingly, miR-195-5p was observed in the intersection (Fig. 3C). As described in a prior study, HDAC3 can bind to the miR-195-5p promoter and inhibit its transcription process [11]. Besides, miR-195-5p has been proposed as an downregulated miRNA that closely correlates with the clinical stage in RCC [12]. Based on TCGA data, we found that HDAC3 expression inversely correlated that of mir-195-5p (Additional file 1: Fig. S1D); therefore, miR-195-5p was chosen for further study.

Subsequent experiments were performed to explore the downstream regulatory mechanism of HDAC3 in RCC. RT-qPCR documented the markedly downregulated expression of miR-195-5p in RCC tissues and A498 cells (Fig. 3D, E) as compared to their corresponding controls. Besides, an inverse modulatory effect of HDAC3 on miR-195-5p expression was identified through the results of Western blot and RT-qPCR assays (Fig. 3F, G). For the putative binding of HDAC3 to the miR-195 promoter, we performed ChIP assay. Based on the results, an enrichment of miR-195 promoter region could be detected in the DNA precipitated by HDAC3 antibody, rather than in the DNA precipitated by matched isotype IgG (Additional file 2: Fig. S2A), indicating the direct interaction between HDAC3 and miR-195 promoter. Since HDAC inhibits gene transcription through histone deacetylation, whether HDAC3 silencing could augment the miR-195 expression via restoring histone acetylation was examined. Our current results showed that when HDAC3 was silenced, the amount of miR-195 promoters precipitated by K27-acetylated histone H3 antibody increased significantly (Additional file 2: Fig. S2B), suggesting an increase in histone H3 acetylation linked to miR-195 promoter.

To determine the regulatory role of HDAC3 and miR-195-5p in RCC, we transfected si-negative control (NC), si-HDAC3, si-HDAC3 + inhibitor NC, or si-HDAC3 + miR-195-5p inhibitor into the A498 cells. RT-qPCR and Western blot data confirmed the transfection efficiency (Fig. 3H). EdU assay demonstrated that the downregulation of HDAC3 suppressed proliferation of A498 cells, which could be rescued when miR-195-5p expression was downregulated simultaneously (Fig. 3I), thus indicating that silencing HDAC3 inhibited the A498 cell proliferation via miR-195-5p. Furthermore, the aforementioned transfected A498 cells were co-cultured with CD3^+^ T cells. The results showed that the downregulation of HDAC3 enhanced CD3^+^ T cell proliferation and elevated the expression of IFN-*γ* released from CD3^+^ T cells, which effect could be suppressed by downregulation of miR-195-5p in combination (Fig. 3J, K). Collectively, HDAC3 facilitated the RCC cell proliferation and suppressed T cell activation via repressing miR-195-5p transcription.

### Upregulated miR-195-5p inhibits RCC cell proliferation and promotes T cell activation by targeting SGK1

**Fig. 4:**
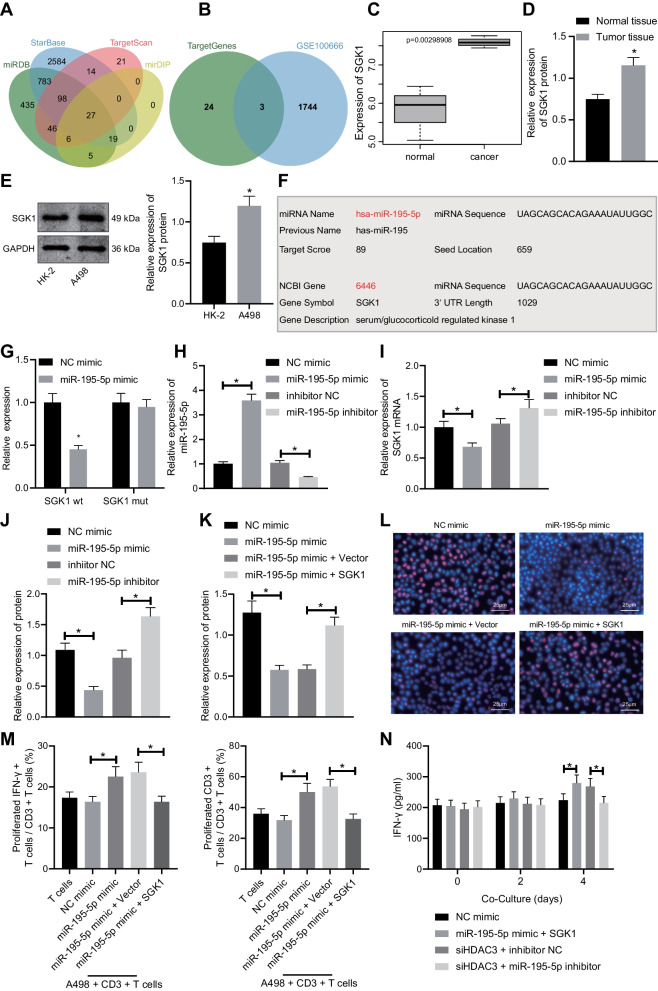
RCC proliferation is curbed, and T cell activation is facilitated by miR-195-5p through targeted inhibition of SGK1. **A** The intersection Venn diagram of downstream target genes of miR-195-5p predicted by miRDB, StarBase, TargetScan, and mirDIP. **B** The intersection Venn diagram of upregulated differentially expressed genes from GSE100666 dataset and candidate target genes. **C** The expression box diagram of SGK1 in RCC cancer samples from the GSE100666 dataset. **D** The expression of SGK1 in RCC and adjacent non-cancerous tissues determined by Western blot analysis (*n* = 58). *p < 0.05 versus the adjacent non-cancerous tissues. **E** The expression of SGK1 in the RCC cell line (A498) and normal renal cell line (HK-2) normalized to GAPDH determined by Western blot analysis. *p < 0.05 versus the HK-2 cell line. **F** Targeting relationship between miR-195-5p and SGK1 determined with TargetScan. **G** Relative luciferase activity measured by the dual-luciferase reporter gene assay. *p < 0.05 versus the NC mimic group*.* **H** miR-195-5p expression in A498 cells determined by RT-qPCR. *p < 0.05 between the two groups*.* **I** mRNA expression of SGK1 in A498 cells after transfection determined by RT-qPCR. *p < 0.05 between the two groups*.* **J** and **K** The protein expression of SGK1 normalized to GAPDH determined by Western blot analysis following different treatments. *p < 0.05 between the two groups*.* **L** A498 cell proliferation detected by EdU assay. *p < 0.05 between the two groups*.* **M** IFN-*γ*^+^ T cell proportion and CD3^+^ T cell proliferation detected by flow cytometry. *p < 0.05 between the two groups*.* **N** The expression of IFN-*γ* released from CD3^+^ T cells determined by ELISA. *p < 0.05 between the two groups*.* The results were measurement data and expressed as mean ± standard deviation. The results were collected from at least 3 independent cell experiments. Data between RCC and adjacent non-cancerous tissues were analyzed by paired *t* test, and data comparison between other the two groups was analyzed by unpaired *t* test

To further investigate the downstream regulatory factors of miR-195, we jointly predicted miRNA target genes through the bioinformatics websites miRDB, StarBase, TargetScan, and mirDIP and ultimately obtained 27 genes in the intersection (Fig. 4A). Subsequently, we obtained 1747 differentially upregulated genes through the differential analysis on the RCC-related dataset GSE100666 and intersected these genes with the 27 candidate target genes, where KCNJ2, CCND1, and SGK1 were obtained (Fig. 4B). A previous study has shown that SGK1 can inhibit T cell immune function and promote tumorigenesis [21]. Moreover, according to prediction results online, a direct binding of miR-195-5p to the 3′UTR of SGK1 was suggested; through the differential analysis on the GSE100666 microarray, SGK1 was found a highly expressed gene in RCC (Fig. 4C). Given these findings, we speculated that the regulatory role of miR-195-5p in RCC might be dependent on SGK1 3′UTR.

For further verification, Western blot analysis was performed to measure the expression profile of SGK1 in RCC tissues and cells (Fig. 4D, E), results of which indicated a significant upregulation of SGK1 in RCC tissues and A498 cells when compared with their corresponding controls. Binding site and sequence between miR-195-5p and SGK1 were attained through TargetScan (Fig. 4F). By constructing luciferase reporter vector and corresponding mutant vector of SGK1 (Fig. 4G), the luciferase assay exhibited data showing the miR-195-5p-targeted regulation of SGK1. Meanwhile, we demonstrated that overexpression of miR-195-5p could upregulate, while silencing of miR-195-5p could downregulate the mRNA and protein expression of SGK1 (Fig. 4H–J).

To further explore the RCC, cell proliferation and immune response affected by miR-195-5p through targeted inhibition of SGK1, NC mimic, miR-195-5p mimic, miR-195-5p mimic +  vector, or miR-195-5p mimic + SGK1-flag were delivered into A498 cells followed by confirmation of transfection efficiency with Western blot analysis. The transfection efficiency was qualified for subsequent experiments (Fig. 4K). EdU assay demonstrated that delivery of miR-195-5p mimic suppressed the A498 cell proliferation, while SGK1 counterweighed the anti-proliferative effects of miR-195-5p (Fig. 4L). Through A498-CD3^+^ T cell co-culture experiments, flow cytometric analysis revealed that overexpressed SGK1 could override the enhanced T cell activation and increased expression of CD3^+^ T cell-released IFN-*γ* induced by miR-195-5p mimic (Fig. 4M, N). Hence, these findings bring us to the consensus that upregulated miR-195-5p curbed RCC cell proliferation and enhanced T cell activation through the target inhibition of SGK1.

### PDCD5 curbs RCC cell proliferation and enhances T cell activation via the HDAC3/miR-195-5p/SGK1 axis

**Fig. 5:**
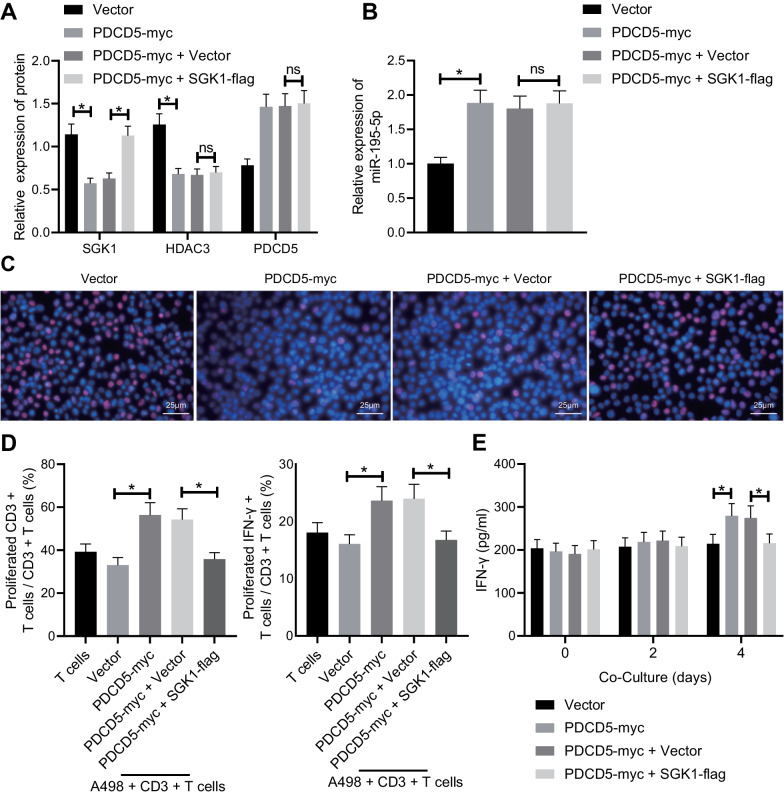
RCC proliferation is restrained, and T cell activation is stimulated by PDCD5 via the HDAC3/miR-195-5p/SGK1 axis. **A** The protein expression of PDCD5, HDAC3, and SGK1 in A498 cells normalized to GAPDH determined by Western blot analysis. **B** miR-195-5p expression in A498 cells determined by RT-qPCR. **C** A498 cell proliferation detected by EdU assay. **D** IFN-*γ*^+^ T cell proportion and CD3^+^ T cell proliferation detected by flow cytometry. **E** The expression of IFN-*γ* released from CD3^+^ T cells determined by ELISA. *p < 0.05 between the two groups. The results were measurement data and expressed as mean ± standard deviation. The results were collected from at least 3 independent cell experiments

The above-mentioned results determined the respective regulatory role of PDCD5, HDAC3, miR-195-5p, and SGK1 in mediating RCC cell proliferation and immune response. Moreover, inverse relations in pairs were also identified. We then endeavored to investigate whether PDCD5 could be functional via the HDAC3/miR-195-5p/SGK1 axis. Hence, vector, PDCD5-myc, PDCD5-myc + vector, and PDCD5-myc + SGK1-flag were transduced into A498 cells followed by quantification using Western blot analysis (Fig. 5A) and RT-qPCR (Fig. 5B). It was found that expression of HDAC3 and SGK1 was diminished, while miR-195-5p expression was elevated in response to PDCD5 overexpression, while SGK1 expression was rescued by treatment with SGK1-flag. Convincingly, a series of assays such as EdU (Fig. 5C), flow cytometry (Fig. 5D), and ELISA (Fig. 5E) offered evidence demonstrating that PDCD5 overexpression inhibited the A498 cell proliferation, induced the CD^3+^ T cell activation, and promoted the T cell immunity, which effects were all reversed after SGK1 re-expression. To summarize, the HDAC3/miR-195-5p/SGK1 axis may participate in the influence of PDCD5 on RCC cell proliferation and T cell activation.

### PDCD5 inhibits in vivo tumorigenicity of RCC cells via the HDAC3/miR-195-5p/SGK1 axis

**Fig. 6:**
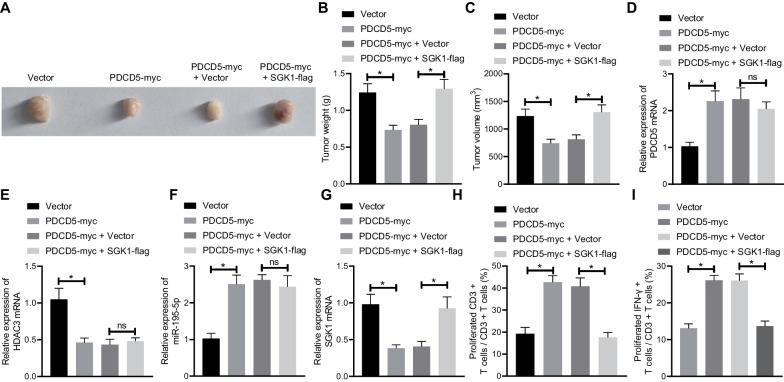
PDCD5 regulates the HDAC3/miR-195-5p/SGK1 axis to inhibit in vivo tumorigenicity of RCC cells. **A** Representative images of resected tumors. **B** Tumor weight. **C** Tumor volume. **D** mRNA expression of PDCD5 in the tumor tissues of nude mice determined by RT-qPCR. **E** mRNA expression of HDAC3 in the tumor tissues of nude mice determined by RT-qPCR. **F** miR-195-5p expression in the tumor tissues of nude mice determined by RT-qPCR. **G** mRNA expression of SGK1 in the tumor tissues of nude mice determined by RT-qPCR. **H**, IFN-*γ*^+^ T cell proportion detected by flow cytometry. **I** The expression of IFN-*γ* released from CD3^+^ T cells determined by ELISA. *n* = 8. *p < 0.05 between the two groups. The results were measurement data and expressed as mean ± standard deviation

After unraveling the regulatory role of PDCD5-mediated HDAC3/miR-195-5p/SGK1 axis in vitro, we developed a mouse model for in vivo validation. A498 cells treated with vector, PDCD5-myc, PDCD5-myc +  vector or PDCD5-myc + SGK1-flag were subcutaneously injected into mice. After 4 to 5 weeks, tumors were resected, measured, and weighed as shown in Fig. 6A–C. Our results exhibited that PDCD5 could significantly weaken the tumorigenicity of A498 cells, which could be reversed by SGK1. Based on RT-qPCR data, the expression of HDAC3 and SGK1 decreased and that of miR-195-5p increased after overexpression of PDCD5, whereas SGK1 expression was elevated in the tumors derived from PDCD5-myc + SGK1-flag-treated cells as compared with those derived from PDCD5-myc + vector-treated cells (Fig. 6D–G). Subsequently, T cells were isolated from tumors, followed by flow cytometry. It was found that high expression of PDCD5 contributed to enhanced T cell activation and increased release of IFN-*γ*, which was abrogated by overexpressed SGK1 (Fig. 6H, I). Briefly, the inhibitory role of PDCD5 in tumorigenicity of RCC cells via the HDAC3/miR-195-5p/SGK1 axis was confirmed in vivo.

## Discussion

**Fig. 7:**
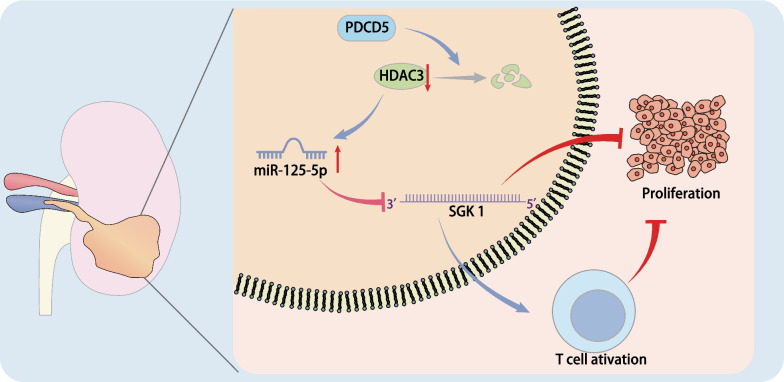
Mechanism map of PDCD5/HDAC3/miR-195-5p/SGK1 axis in the progression of RCC. PDCD5 harbors tumor-suppressive property in RCC by inhibiting the proliferation of RCC cells and enhancing the immune functions of T cells via the HDAC3/miR-195-5p/SGK1 axis

Clinical review has depicted the significance of therapeutic strategies based on immunomodulation to the management of RCC [22]. Thus, in this present study, we attempted to uncover the regulatory role of PDCD5 in the development and progression of RCC regarding immune response. Collectively, our experimental data demonstrated that PDCD5 harbored tumor-suppressive property by inhibiting the proliferation of RCC cells and enhancing the immune functions of T cells via the HDAC3/miR-195-5p/SGK1 axis (Fig. 7).

Fundamentally, downregulation of PDCD5 and high expression of HDAC3 were found in RCC tissues and cells followed by identification of an inverse relation between PDCD5 and HDAC3. Upregulated PDCD5 or downregulated HDAC3 inhibited the RCC cell proliferative potential and promoted the T cell activation, corresponding to an upregulated IFN-*γ* release from T cells. The anti-tumor properties of PDCD5 have been reported in mice with skin cancer by contributing to apoptosis [23]. Furthermore, downregulated PDCD5 is indicative of dismal pathological features of patients with ovarian cancer [24]. Besides, PDCD5 expression has been observed to be significantly reduced in highly differentiated lung adenocarcinoma, whereas the re-expression of PDCD5 exerts anti-tumorigenic effects by curbing malignant cell proliferation [25]. In addition to its implication in tumorigenesis, PDCD5 has been reviewed to be functional in the mediation of inflammation [26], and immunological functions [27]. Nevertheless, there lacks supportive evidence that links the PDCD5 expression to T cell function in RCC prior to this study. Moreover, PDCD5 mediates the activation of p53 by degrading HDAC3, thus playing a crucial role in the gastric carcinogenesis [20]. As key regulators of histone acetylation, HDAC can remove the acetyl group of histone lysine residue, resulting in the re-establishment of positive charge in histone, chromatin compactness, and transcriptional inhibition [28]. Intriguingly, HDAC3, one of the most characteristic HDACs, is dysregulated in multiple types of cancers, such as liver cancer [29] and colorectal cancer [30]. Intriguingly, a previously reported study has shown an upregulation of HDAC3 in tumor tissues of RCC [31]. In consistent with our findings, high expression of HDAC3 has also been identified in colorectal tumors in association with tumor differentiation grade [30]. Increasing studies have highlighted that the anticancer effects of small molecule drug inhibition by HDAC inhibitors, such as SAHA and MS-275, can induce tumor cell apoptosis and impair tumor invasion and angiogenesis [32, 33]. Notably, HDAC3 inhibitors are considered as a promising candidate for immunity-based therapy in pancreatic cancer [34]. In this study, we unraveled that siRNA-mediated genetic silencing of HDAC could repress RCC cell proliferation and motivate T cell activation, contributing to its anti-tumor activity. However, the anticancer mechanism of HDAC inhibition remains to be fully clarified. In the current study, repression of HDAC3 by RNA interference significantly induced the expression of miR-195-5p, suggestive of miR-195 as a downstream anticancer target. Further mechanistic investigations revealed that the regulatory mechanism of PDCD5/HDAC3 was dependent on the miR-195-5p-mediated targeting of SGK1. Concordantly, the results in our study showed that miR-195-5p was downregulated in RCC, while downregulation of miR-195-5p counterweighed the anti-tumor action of silencing HDAC3. The tumor-suppressive property of miR-195 has been documented in clear cell RCC since re-expressed miR-195 leads to weakened malignant phenotypes [35]. In partial line with our findings, enforced expression of miR-195 has been suggested to elevate the release of IFN-*γ* from T cells to alleviate immune evasion of diffuse large B cell lymphoma through downregulation of programmed death-ligand 1 [36]. The production of IFN-*γ* has also been demonstrated to be repressed by SGK1 activation in T cells [21]. The fact that SGK1 is a critical mediator of transepithelial sodium transport through activation of the epithelial sodium channel in renal tubules [37, 38] is highly suggestive of its involvement in kidney-related disorder and malignancies. Moreover, SGK1 activation is deciphered to have a pivotal role in controlling the growth of renal cancer cells via IL-2 mediation [39]. It is noteworthy that the implication of PDCD5/HDAC3/miR-195-5p/SGK1 axis in RCC has been rarely studied through their respective interaction which has been briefly mentioned in other cancers. PDCD5 can induce HDAC3 cleavage and ubiquitin-dependent proteasome degradation, which is relevant to unfavorable prognosis of gastric cancer patients [20]. It has been reported that HDAC3 inhibitor gives rise to cell cycle arrest and apoptosis induction in RCC [31]. miR-195-5p has also shown its tumor-inhibiting activity in RCC [12]. Importantly, a prior study has proposed the inverse relation between HDAC3 expression and miR-195 expression in human HCC tissues as well as their binding relation in HCC [11]. Here, our experimental results show that PDCD5 may promote the degradation of HDAC3, upregulate the expression of miR-195-5p, and inhibit the expression of SGK1, which promotes T cell activation and inhibits the proliferation of RCC cells, thereby preventing the occurrence and development of RCC. Whether this axis also functions in other cancers in addition to RCC is still an interesting topic to be discussed in future.

## Conclusion

Taken together, our findings provided a novel mechanistic insight to improve the understanding of the role of PDCD5 in immunomodulation of tumorigenesis and revealed PDCD5 as a potential target for therapeutic intervention against RCC via the HDAC3/miR-195-5p/SGK1 axis. However, further studies are required for the development of immunotherapeutic regimens to trigger the strong response to IFN-*γ*, and thus, durable anti-tumor responses could be developed following immune interference at the initial stage [40]. Moreover, due to the complex microenvironments and interaction, further research is required to better illuminate the reported regulatory mechanism in detail for its translation into clinical practice. Therefore, we hope to use miRNA maps, RNA sequencing, and innovative path analysis to predict the target of miRNA, explore the correlations of PDCD5, HDAC3, miR-195-5p, and SGK1 expression patterns in clinical samples with clinical parameters, and investigate whether activation and recruitment of other immune cells have synergistic response during the process.

## Materials and methods

### Ethics statement

The study was performed with the approval of the Ethics Committee of the University of South China. All participants signed written informed consent before our investigation. The experimental animals were treated according to the Guide for the Care and Use of Laboratory Animals.

### Bioinformatics analysis

RCC-related dataset GSE100666 was obtained through the GEO database, which contained 3 normal tissue samples and 3 RCC cancer tissue samples. From this dataset, differentially expressed genes were selected using the “limma” package in R language with the thresholds of |logFC|> 1 and *p* < 0.05. The differential expression of HDAC3 in pan-cancer was predicted using a bioinformatics website StarBase, and the downstream regulatory miRNAs of HDAC3 were obtained using TransmiR v2.0 and RNAInter, followed by intersection using the jvenn tool. Additionally, RCC-related miRNAs were retrieved through the GeneCards database and screened based on the correlation score, and the screened ones were intersected with the aforementioned downstream regulatory miRNAs of HDAC3. Next, the miRNA target genes were predicted using miRDB, StarBase, TargetScan, and mirDIP tools jointly and the acquired genes were intersected using the jvenn tool. The differentially expressed genes from GSE100666 dataset were then intersected with the candidate miRNA target genes using the jvenn tool.

### Clinical sample collection

Paired RCC tissues and adjacent non-cancerous tissues (at a distance > 5 cm from tumor tissues) were resected from 58 RCC patients (40 males, 18 females, mean age: 47.03 ± 8.71 years ranging between 35 and 70 years). The enrolled patients were not treated by chemotherapy or radiotherapy prior to surgical resection. Patients with infectious diseases, autoimmune diseases, or multiple primary carcinomas were excluded.

### Isolation and identification of CD3 + T cells

The serum was removed through centrifugation supplemented with heparin anticoagulant. A triple volume of red cell lysis buffer was added to lyse the blood cells for 10 min. The supernatant was removed following 5-min centrifugation. The cells were then rinsed by 5 mL sterile phosphate-buffered saline (PBS) followed by removal of the supernatant through centrifugation. Afterward, cells were re-suspended in sterile PBS before cell counting. Flow cytometric analysis was followed to sort out PE-CD3^+^ T cells (130-113-129, 1: 50, Miltenyi Biotec Technology & Trading, Bergisch Gladbach, Germany).

### Co-culture of RCC cells and T cells

The CD3^+^ T cells (10^5^ cells/96-well plate) tagged by 5,6-carboxyfluorescein diacetate succinimidyl ester (CFSE) were co-cultured with the transfected RCC cells in the medium replenished with recombinant human interleukin-2 (rhIL-2) (20 IU/mL), anti-CD3 (2 μg /mL), and anti-CD28 (1 μg/mL). 48 h later, flow cytometric analysis was conducted.

### Cell culture

The cells applied for cell experiments included RCC cell lines, Caki-1 (cultured with McCoy’s 5a Medium Modified + 10% fetal bovine serum [FBS]), A498 (cultured with Dulbecco’s Modified Eagle’s medium [DMEM] + 10% FBS), 786-O (cultured with Roswell Park Memorial Institute [RPMI]-1640 + 10% FBS), and 769-P (cultured with RPMI-1640 + 10% FBS), as well as normal renal cell line HK-2 (cultured with keratinocyte serum-free medium + 0.05 mg/mL bovine pituitary extract + 5 ng/mL epidermal growth factor) from American Type Culture Collection (Rockefeller, Maryland, USA). Cell culture was performed in an incubator at 37 °C with 5% CO_2_.

### Reverse transcription-quantitative polymerase chain reaction (RT-qPCR)

Tissue and cellular total RNA extraction was conducted utilizing the miRNeasy Mini Kit (QIAGEN, GmbH, Hilden, Germany) and TRIzol reagent (15,596,026, Invitrogen, Carlsbad, CA, USA), respectively. NanoDrop ND-1000 Spectrophotometry (NanoDrop Products, Wilmington, DE, USA) was introduced to measure the RNA quality and concentration. For mRNA detection, complementary RNA (cDNA) was obtained by reverse transcription using reverse transcription kits (RR047A, Takara, Japan). For miRNA detection, the cDNA of miRNA containing polyA tails was obtained employing polyA-tailing detection kits (b532451, Sangon, Shanghai, China) which contained miRNA universal PCR reverse primer and U6 universal PCR reverse primer. Primer sequences were synthesized by Sigma-Aldrich (St Louis, MO, USA), which are presented in Additional file 3: Table S1. U6 was selected as an internal reference for miR-195-5p, while glyceraldehyde 3-phosphate dehydrogenase (GAPDH) was applied as an internal reference for SGK1 and HDAC3. Their relative transcription levels were calculated employing a relative quantitative method (the 2^−△△Ct^ method) [41].

### Western blot analysis

Total protein isolated from cells and tissues was lysed in 100 µL of lysis buffer at 4 °C for 30 min, followed by 20 min of centrifugation at 12,000 r/min, 4 °C to collect the supernatant. The protein concentration measurement was realized with a bicinchoninic acid kit (20201ES76, Yeasen Biotech Co., Ltd., Shanghai, China). Following sodium dodecyl sulfate–polyacrylamide gel electrophoresis, the isolated proteins were loaded onto a nitrocellulose filter membrane. The protein-carried membrane was blocked with 5% skimmed milk powder at 4 °C overnight and further probed with specific primary antibodies at 4 °C overnight, including rabbit anti-mouse antibodies to PDCD5 (ab75430, 1: 1000, Abcam Inc., Cambridge, UK), HDAC3 (ab32369, 1: 1000, Abcam), SGK1 (ab32374, 1: 500, Abcam), and CD3 (ab16669, 1: 25, Abcam). The membrane was re-probed with diluted horseradish peroxidase-marked goat anti-rabbit immunoglobulin G (IgG) antibody (ab6721, 1: 5000, Abcam) at ambient temperature for 1 h. After visualization, development, and photographic fixing, gray values of protein bands were analyzed by Quantity One software followed by quantitative protein analysis with GAPDH (ab181602, 1: 5000, Abcam) as the internal reference.

### Coimmunoprecipitation (co-IP) assay

The A498 cells were lysed in NP-40 buffer (consisting of 25 mm Tris–HCl (pH 7.4), 150 mM NaCl, 1 mm EDTA, 5% glycerol, and 1% NP-40) which contained a complete EDTA-free protease inhibitor mixture (Roche Life Science). Next, the cell extract was cultured overnight at 4 °C with allogeneic rabbit anti-IgG (ab172730, Abcam) or PDCD5 antibody (ab75430, Abcam) or HDAC3 (ab32369, Abcam)-coupled protein G beads (10004D, Roche Life Science). Finally, immune complexes were harvested for Western blot analysis.

### Chromatin immunoprecipitation (ChIP) assay

A498 cells were non-treated or manipulated with siRNA for 48 h, cross-linked with formaldehyde, and then subjected to cell lysis and ultrasonic treatment. Chromatin complexes were immunoprecipitated with the use of anti-HDAC3, K27-acetylated histone H3 (Ab4729, Abcam), or homotyped matched anti-IgG (as NC). Following incubation with Dynabeads protein G beads (10004D, Life Technologies), the complexes were harvested. The cross-linking of DNA protein was reversed by heating. After DNA purification, semi-qPCR amplification was performed using the primers covering the HDAC3-binding site within the miR-195-5p promoter. Genomic regions lacking HDAC3-binding sites upstream of GAPDH were used as controls. The qPCR amplification was conducted for purified DNA analysis on LightCycler 480 (Roche Life Science). The input DNA was utilized for normalization of antibody-immunoprecipitated DNA. The primer sequences were: F: CTGGAGCAGCACAGCCAATA, R: AGCTTCCCTGGCTCTAGCA.

### 5-Ethynyl-2′-deoxyuridine (EdU)

The cells were fixed with 4% paraformaldehyde for 15 min and permeabilized in 1% Triton X-100 for 20 min, followed by incubation with 10 μmol/L EdU (Life Technologies) for 1.5 h. Cells were incubated with the culture medium supplemented with click-it reaction mixture at room temperature for 30 min, stained with Hoechst 33,342 for 30 min, and observed under a fluorescence microscope (Olympus). The cell proliferation rate was obtained based on the proportion of EdU-positive cells.

### Cell transfection

Cells were seeded into a 6-well plate for 24 h. After achieving 70% of confluence, 20 μL Lipofectamine 2000 (11,668,019, Thermo Fisher Scientific) was diluted in 500 μL serum-free medium and allowed to stand for 5 min. Meanwhile, the plasmids and liposomes were gently mixed and allowed to incubate together for 20 min. The cells were washed by serum-free medium for 3 times. Every 2 mL serum-free medium was supplemented into each well, and a liposome mixture was added for another incubation for 5–24 h. Following 3 times of washing by serum-free medium, cells were cultured in 20% DMEM without antibiotics for 48 h. The medium was renewed 6 h post transfection. The cell culture lasted for 48 h before their use for subsequent experiments.

### Dual-luciferase reporter gene assay

The 3′-untranslated region (3′-UTR) of SGK1 wild type (WT) and mutant (MUT) was artificially synthesized. After restriction enzyme digestion on pmiR-RB-REPORT™, gene fragments WT and MUT were, respectively, inserted into the pmiR-RB-REPORT™ vector (which was constructed by Guangzhou RiboBio Co., Ltd., Guangzhou, Guangdong, China). WT and MUT reporter plasmids together with NC mimic or miR-195-5p mimic were introduced into HEK293T cells, while blank vectors served as controls. 48 h following treatment, the supernatant of cell lysate was harvested through centrifugation for 3–5 min. With the application of the Dual-Luciferase Reporter Assay Kit (YDJ2714, Shanghai Yuduo Biotech Co., Ltd., Shanghai, China), luciferase activity was analyzed in the Dual-Luciferase Reporter Assay System (Promega, Madison, WI, USA). The relative luciferase activity = relative light unit (RLU) of firefly luciferase/RLU of Renilla luciferase.

### Cell surface antigen detection

Following dispersion into a single cell suspension, the collected cells were re-suspended in binding buffer (BD Biosciences). Before use, pacific blue-interferon-*γ* (IFN-*γ*) (BioLegend, #505,817, Rat, 1: 50) was subjected to fixation and permeabilization. The pacific blue-IFN-*γ* or PerCP-CD3 (BioLegend, #100,326, Armenian Hamster, 1: 100) was added into T cells, followed by detection on a BD FACS Canto II flow cytometer (BD Immunocytometry Systems, San Jose, CA, USA) as well as analysis using Flow Jo software.

### *CD3* + *T cell proliferation assessment*

The 96-well plate was enveloped with a tetramer antibody of anti-CD3/anti-CD28 (STEMCELL Technologies, Vancouver, Canada). CD3^+^ T cells were stimulated with IL-2 (20 IU/mL) and then tagged with CFSE (S1076, Solarbio Co., Ltd., Beijing, China) before co-incubation with RCC cells in RPMI-1640 medium at 37 °C with 5% CO_2_. The cells with low CFSE signals shown by flow cytometric analysis were regarded as proliferated cells.

### Enzyme-linked immunosorbent assay (ELISA)

The content of IFN-*γ*, released from T cells, was measured in strict accordance with the manual of ELISA kits (JLC5013, Jingkang Bioengineering Co., Ltd., Shanghai, China).

### Xenograft tumor in nude mice

A498 cells (10^6^) were re-suspended in 100 µL normal saline and subcutaneously delivered into the armpit of nude mice with immunodeficiency (*n* = 8). The tumor volume measurement was implemented every 2 days. The tumors were resected and imaged before RT-qPCR and cell surface antigen detection.

### Statistical analysis

All experimental data were analyzed using the SPSS 21.0 software (IBM Corp. Armonk, NY, USA). Measurement data were summarized as mean ± standard deviation. Following normal distribution and homogeneity of variance, data between two groups in paired design were compared by paired *t *test and those in unpaired design were analyzed by unpaired *t *test. Multigroup comparisons were performed by one-way analysis of variance (ANOVA) with assistance of Tukey’s post hoc test. Data among multiple groups at various time points were analyzed employing Bonferroni-corrected repeated measures ANOVA. A value of *p* < 0.05 was deemed as statistically significant.

## Supplementary Information

**Additional file 1**: **Fig. S1**. PDCD5 can interact with HDAC3 while HDAC3 expression negatively correlates with miR-195-5p expression. **A** The significant negative correlation between PDCD5 expression and HDAC3 expression (*r* = − 0.27, *p* < 0.001) predicted by the TCGA database. **B** In class I HDAC, PDCD5 selectively interacts with HDAC3 (proteins from A498 whole cell lysates are immunoprecipitated and then immunoblotted with the indicated antibodies). **C** Cells were treated with MG132 for 3 h, followed by immunoprecipitation, and HDAC3 ubiquitination level was measured by Western blot assay using anti-ubiquitin antibody. **D** The significant correlation between HDAC3 and miR-195-5p (*r* = − 00.237, *p* < 0.001) predicted by the TCGA database.

**Additional file 2**: **Fig. S2**. HDAC3 binds to miR-195 promoter. **A** The binding of HDAC3 to miR-195 promoter region as detected by ChIP assay on chromatin extract of A498 cells using anti-HDAC3 antibody or control (IgG). **B** A498 cells were transfected with si-NC or si-HDAC3 for 48 h, and then ChIP assay was performed with antibodies against K27-acetylated histone H3 (anti-acH3) or control (IgG). *p < 0.05 between the two groups. The cell experiment was repeated independently three times.

**Additional file 3**: **Table S1**. Primer sequences for RT-qPCR.

## Data Availability

The datasets generated/analyzed during the current study are available.

## Abbreviations

RCC: Renal cell carcinoma
HDAC3: Histone deacetylase 3
miR-195-5p: MicroRNA-195-5p
SGK1: Serum glucocorticoid-inducible kinase 1
IFN-*γ*: Interferon-*γ*
PDCD: Programmed cell death
PBS: Phosphate-buffered saline
CFSE: 5,6-Carboxyfluorescein diacetate succinimidyl ester
RT-qPCR: Reverse transcription-quantitative polymerase chain reaction
cDNA: Complementary RNA
GAPDH: Glyceraldehyde 3-phosphate dehydrogenase
IgG: Immunoglobulin G
co-IP: Coimmunoprecipitation
ChIP: Chromatin immunoprecipitation
EdU: 5-Ethynyl-2′-deoxyuridine
3′-UTR: 3′-Untranslated region
WT: Wild type
MUT: Mutant
RLU: Relative light unit
ELISA: Enzyme-linked immunosorbent assay
ANOVA: Analysis of variance
