# Supplementary material for: PDCD5 inhibits progression of renal cell carcinoma by promoting T cell immunity: with the involvement of the HDAC3/microRNA-195-5p/SGK1
Source: Clin Epigenetics. 2022 Oct 20;14:131. doi: 10.1186/s13148-022-01336-1 (PMC9583501; doi:10.1186/s13148-022-01336-1)
Supplement: Supplementary file 3 — Additional file 3: Table S1. Primer sequences for RT-qPCR. [file 13148_2022_1336_MOESM3_ESM.docx]

**Table S1** Primer sequences for RT-qPCR

| Gene | Primer sequences (5' - 3') | |
| --- | --- | --- |
| GAPDH | F: TGATGGGTGTGAACCACGAG | R: TCAGTGTAGCCCAAGATGCC |
| U6 | F: GCTTCGGCAGCACATATACTAAAAT | R: Reverse universal primer |
| SGK1 | F: GGAGATTGGCCGTATCCCAC | R: GGTGTACTTCAGGCTGGGAC |
| HDAC3 | F: CACCCTATGAAGCCCCATCG | R: GAGACCGTAATGCAGGACCAG |
| miR-195-5p | F: TAGCAGCACAGAAATATTGGC | R: Reverse universal primer |

Note: RT-qPCR, reverse transcription quantitative polymerase chain reaction; F, forward; R, reverse; GAPDH, glyceraldehyde-3-phosphate dehydrogenase; SGK1, serum glucocorticoid-inducible kinase 1; HDAC3, histone deacetylase 3; miR-195-5p, microRNA-195-5p.
